# Gln27Glu variant of Beta2-adrenoceptor gene affects male type fat accumulation in women

**DOI:** 10.1186/1476-511X-8-43

**Published:** 2009-10-15

**Authors:** Tarja Kunnas, Riikka Lahtio, Marja-Leena Kortelainen, Anne Kalela, Seppo T Nikkari

**Affiliations:** 1 Department of Medical Biochemistry, University of Tampere Medical School, Tampere, Finland; 2 Department of Forensic Medicine, University of Oulu, Oulu, Finland; 3 Centre for Laboratory Medicine, Tampere University Hospital, Tampere, Finland

## Abstract

**Background:**

The β_2_-adrenergic receptor (BAR2) is the main lipolytic receptor in white human adipose tissue. There is a functional glutamine 27 glutamic acid (Gln27Glu, rs 1042714) polymorphism in its gene, which has been variably associated with body mass index. This gene variant may be associated with male-type adiposity in women and thus increased cardiovascular risk. We investigated whether the BAR2 Gln27Glu polymorphism is associated with visceral fat and coronary intima thickness in women.

**Methods:**

The amount of mesenteric and omental fat was directly measured and anthropometric measurements were done from 112 forensic autopsy cases of women aged 15 to 49 years. The thickness of the coronary intima, which reflects the severity of atherosclerosis, was measured by computerized image analysis. The BAR2 Gln27Glu polymorphism was determined by polymerase chain reaction.

**Results:**

We found that the amount of visceral fat was significantly higher in women with the Glu allele (689 ± 555 g) compared to Gln/Gln homozygotes (481 ± 392 g, P = 0.023). The waist-hip ratio also tended to be higher in women with the Glu allele compared to Gln/Gln homozygotes (p = 0.050). There were no statistically significant differences between the genotype groups in BMI or the thickness of coronary intima.

**Conclusion:**

The Glu allele of the BAR2 gene may be a risk factor for visceral fat accumulation in young to middle-aged women. However, this polymorphism was not associated with preclinical atherosclerosis.

## Background

Obesity is a rapidly growing public health problem. As men become obese, they usually accumulate fat in the visceral (intra-abdominal) region, which is referred to as male-type fat. As women become obese, fat may accumulate around the hips and thighs, as female-type fat, or as male-type visceral fat. Male-type obesity is associated with cardiovascular disease, insulin resistance and type 2 diabetes, particularly among women [1,2]. Visceral adiposity also increases overall disease risk even in those who are not obese [3].

Genetic factors have been shown to be important in fat distribution [4]. The β_2_-adrenergic receptor (BAR2) is the main lipolytic receptor in white adipose tissue [5]. Several functional polymorphisms occur within the coding region of this gene. The glutamine 27 glutamic acid (Gln27Glu, c79C>G, rs 1042714) polymorphism has been considered an important factor in overall obesity [6], although conflicting results also exist [7-9]. Interestingly, the Glu27 allele has been associated with visceral obesity, quantified by computed tomography [10].

Since the β_2_-adrenergic receptor plays an important role in the parasympathetic and sympathetic regulation of heart rate and contractility both in intact cardiovascular system and in disease, different polymorphisms may have an effect on the cardiovascular function [11]. The contribution of a single gene polymorphism is expected to manifest itself in younger - as in our study of young to middle-aged women - rather than in older patients. Thus, the Gln27Glu polymorphism has been suggested to be an independent risk factor for cardiovascular diseases [12-15].

The present study was performed to investigate whether the Gln27Glu polymorphism is associated with accumulation of visceral adipose tissue, and whether it is an explanatory factor for the male-type obesity in young to middle-aged women. In addition, we examined whether the intima thickness explains the suggested role of Gln27Glu polymorphism in cardiovascular diseases.

## Methods

### Subjects

The material was collected from medico-legal autopsies of sudden deaths, at the Department of Forensic Medicine, University of Oulu, Finland. A total of 112 women from 15 to 49 years of age (median 37 years) were examined. The subjects had died of coronary disease (n = 5), other diseases of the circulatory system (n = 23), external causes (n = 82), cancer (n = 3), and other classified causes (n = 9). The study protocol was approved by the Ethics Committee of the University of Oulu.

### Anthropometric measurements

Height (cm), weight (kg), waist circumference (cm), hip circumference (cm), and the amount of mesenteric and omental fat (g) were measured as described previously [16]. Body mass index (BMI; kg/m2) and waist-to-hip ratio (WHR) were calculated from these measurements.

### Morphometric measurements in coronary artery samples

The left and right coronary arteries were opened longitudinally and excised free from the heart and fixed in 10% neutral formalin for 24 h. For morphometric analysis, five transversely cut samples were taken from the most lesion-occupied regions of the anterior descending artery (LAD), circumflex artery (CX) and right coronary artery (RCA). The samples were taken from the proximal parts of the vessels when there were no visible lesions [14]. The paraffin-embedded samples were sectioned transversely at 5 μm and stained using the Verhoeff-Masson trichrome method. In coronaries with diffuse intimal thickening, the thickness of the intimal layer was measured taking the internal elastic lamina as a border between intima and media. In the samples with more advanced lesions, the internal elastic lamina often showed variable degrees of destruction and the maximum thickness of the intima was measured from the limit of the intimal and medial layer. The measurements were made at 40× magnification by using computerized image analysis (Image Tool; University of Texas Health Science Center in San Antonio, USA). For each vessel (LAD, CX, and RCA) the results represent the means of five measurements at different sites. The highest intima thickness, whether it was in LAD, CX, or RCA, was used for each case as the dependent variable for statistical analysis.

### DNA extraction and BAR2 genotyping

Genomic DNA was extracted from autopsy samples using a commercial kit (Qiagen Inc., Valencia, Calif., USA). The following primers were used in polymerase chain reaction: forward 5' GAA TGA GGC TTC CAG GCG TC 3'/reverse 5' GGC CCA TGA CCA GAT CAG CA 3. The amplified product was digested with SatI (Fermentas Inc. U.S.A) and the fragments were separated using 1.5% agarose gel.

### Statistical analysis

Statistical analyses were carried out using SPSS 14.0.1 for Windows (SPSS Inc., Chicago, Illinois, USA) using independent-samples t-test or analysis of covariance (ANCOVA) with age and BMI as covariates. Due to skewed distributions, the values for intimal thickness and the amount of mesenteric and omental fat were log-transformed prior to the analyses, although they are presented in crude form. Data are presented as mean values ± SD. Hardy-Weinberg equilibrium was assessed by Chi-Square test.

## Results

The overall frequencies for the genotype groups in the study population were 0.33 for Gln/Gln, 0.54 for Gln/Glu, and 0.13 for Glu/Glu. The genotypes were in Hardy-Weinberg proportions. Since there were only 15 women in the Glu/Glu genotype group and none of the background characteristics differed between genotypes Gln/Glu and Glu/Glu (data not shown), they were combined for further analysis. The phenotypic characteristics of the genotypes are presented in Table 1. The amount of mesenteric and omental fat was significantly higher in women with the Glu allele (689 ± 555 g) compared to Gln/Gln homozygotes (481 ± 392 g, p = 0.023). The difference remained significant even after adjusting for age and BMI (p = 0,030). The waist-hip ratio also tended to be higher in women with the Glu allele compared to Gln/Gln homozygotes (p = 0.050). There was a trend of higher waist circumference in women with the Glu allele compared to Gln/Gln homozygotes (p = 0.094). BMI values and coronary artery intima thicknesses did not differ between the genotypes.

**Table 1:** Phenotype characteristics of the genotypes.

**Genotype**	**Gln/Gln**	**Gln/Glu+Glu/Glu**	*p-value
**N**	**36**	**76**	
Age	33.4 ± 9.9	34.5 ± 10.7	0.595
Body mass index (kg/m^2^)	23.5 ± 5.7	24.8 ± 5.6	0.272
Waist circumference	77.2 ± 14.8	82.3 ± 15.1	0.094
Waist-to-hip ratio	0.83 ± 0.06	0.85 ± 0.09	**0.050**
Mesenteric and omental fat (g)	481 ± 392	689 ± 555	**0.026**
Intima thickness (μm)	468 ± 332	504 ± 377	0.635

*Statistical significance was based on independent-samples t-test.

We also found that all five women who had died of coronary causes had the Glu allele (Gln/Glu genotype). These women had a mean coronary artery intima thickness of 892 ± 185 μm. The risk for death by coronary cause was highly significantly increased in women carrying Glu-allele, compared to the Gln/Gln genotype carriers (p < 0.001).

## Discussion

Our study is the first to show that Glu27 allele of Gln27Glu polymorphism of the BAR2 gene is associated with directly measured mesenteric and omental fat in young to middle-aged women. Our results are in line with those of Lange et al., who reported that the Glu27 variant is associated with visceral obesity [10].

Several studies have found an association between Glu27 allele and obesity among women [6,10,17,18]. However, an association with BMI was not observed in either Austrian, French or Spanish women [19-21]. These controversial results suggest that there may be several modifiers which affect the relation between Gln27Glu polymorphism and obesity. As outlined above, one modifier might be the ethnic group, which has been confirmed in meta-analysis of published studies [22]. Also gender may modify the impact of Gln27Glu polymorphism on obesity, since a study by Hellström et al. showed a positive association between obesity and Glu27 variant in women, but the findings in men were opposite [7]. Then again, the Gln27Glu polymorphism was linked to obesity among Spanish and Japanese men [21,23].

Gene-gene or gene-environmental interactions are likely to mediate the association between Glu27 allele and obesity. Individual responses to different dietary interventions or physical activity may also be genotype dependent. In fact, high carbohydrate intake has been suggested to increase the obesity risk in women carrying the Glu27 allele [24], and both lipolysis and fat oxidation were blunted in obese females during exercise in the Glu27Glu group [17].

The Glu allele has been suggested to be an independent predictor of severe coronary artery disease (CAD) [14]. However, the Cardiovascular Health Study showed that although the Gln27Glu polymorphism was associated with coronary disease, there was no association with carotid intima-media thickness, a marker of preclinical atherosclerosis [25]. Only five of the subjects of the present study had died of CAD. For the others, the maximal coronary artery intima thickness served as a marker of preclinical atherosclerosis. Our results of no association of coronary artery intima thickness with the Gln27Glu polymorphism are therefore in line with the Cardiovascular Health Study.

An interesting aspect of our study is that all five patients of the cohort that had died from CAD carried the Glu-allele and had a mean intimal thickness nearly double that of the remainder of the cohort. However, this finding needs to be documented in a larger population with additional parameters of cardiovascular health, to confirm whether the greater CAD death risk was related to the presence of the Glu-allele and not another risk factor shared by the five. In addition to the small sample size, another limitation of the study is that all the 112 women of the cohort had died of sudden death, which represents a selection bias.

In conclusion, the Glu allele of the Gln27Glu polymorphism was associated with directly measured visceral and omental fat in young to middle-aged women. We suggest that this genetic variation may predispose to visceral adiposity. Instead, no association was found between Gln27Glu polymorphism and preclinical atherosclerosis. This implies that the previously reported association of this gene with CAD may be mediated by gene-environment interactions.

## Competing interests

The authors declare that they have no competing interests.

## Authors' contributions

TK and STN had substantial contributions to conception and design, statistical analysis and writing the manuscript. RL carried out the genotyping. M-L.K did all autopsy measurements and collected the data. AK participated in writing the manuscript. All authors read and approved the final manuscript.
